# Interactions of Linearly Polarized and Unpolarized Light on Kiwifruit Using Aquaphotomics

**DOI:** 10.3390/molecules27020494

**Published:** 2022-01-13

**Authors:** Damenraj Rajkumar, Rainer Künnemeyer, Harpreet Kaur, Jevon Longdell, Andrew McGlone

**Affiliations:** 1 Department of Physics, University of Otago, Dunedin 9016, New Zealand; jevon.longdell@otago.ac.nz; 2 The New Zealand Institute for Plant and Food Research Limited, Ruakura 3216, New Zealand; Rainer.Kunnemeyer@plantandfood.co.nz (R.K.); harpreet.kaur@plantandfood.co.nz (H.K.); Andrew.McGlone@plantandfood.co.nz (A.M.); 3 The Dodd Walls Centre for Photonic and Quantum Technologies, University of Otago, Dunedin 9054, New Zealand

**Keywords:** aquaphotomics, water, spectra, band assignment, functionality, polarization, kiwifruit, near infrared spectroscopy

## Abstract

Near infrared (NIR) spectroscopy is an important tool for predicting the internal qualities of fruits. Using aquaphotomics, spectral changes between linearly polarized and unpolarized light were assessed on 200 commercially grown yellow-fleshed kiwifruit (*Actinidia chinensis* var. *chinensis* ‘Zesy002’). Measurements were performed on different configurations of unpeeled (intact) and peeled (cut) kiwifruit using a commercial handheld NIR instrument. Absorbance after applying standard normal variate (SNV) and second derivative Savitzky–Golay filters produced different spectral features for all configurations. An aquagram depicting all configurations suggests that linearly polarized light activated more free water states and unpolarized light activated more bound water states. At depth (≥1 mm), after several scattering events, all radiation is expected to be fully depolarized and interactions for incident polarized or unpolarized light will be similar, so any observed differences are attributable to the surface layers of the fruit. Aquagrams generated in terms of the fruit soluble solids content (SSC) were similar for all configurations, suggesting the SSC in fruit is not a contributing factor here.

## 1. Introduction

Preference for high-quality fruit has propelled the development of non-destructive techniques for postharvest fruit grading. Although the final decision before purchasing fruit depends upon consumers, the development of instruments used to mimic human judgement is important [1]. One such technique uses near-infrared spectroscopy (NIRS), which has seen success in predicting fruit internal qualities such as soluble solids content (SSC) and dry matter content (DMC) by utilizing multivariate and statistical analyses such as principal components analysis (PCA) and partial least squares regression (PLSR) to create prediction models [2]. Nevertheless, there is still a requirement for improvement of the technique in several areas such as calibration robustness for fruits harvested from different seasons and calibration model transfer between instruments [3,4].

Usually, unpolarized light is used in NIRS. Crossed polarizers in the incident and detection light path are sometimes applied to reduce the amount of specular reflection received [5]. However, polarized light may offer advantages as suggested by some recent studies on pears and apples [6,7].

Implementing polarized light in NIR spectroscopy is simple but, nonetheless, there are few reported studies using polarized light to assess internal quality of fruits, probably because of multiple scattering in the biological tissue, which quickly depolarizes any initially polarized radiation [8]. The degree of polarization for circularly and linearly polarized light has been observed to decrease as green ‘Conference’ pears mature and has been attributed to starch, a polarization sensitive structure, hydrolyzing into smaller sugar molecules, resulting in a decrease in reduced scattering [6]. Advancements in polarized light interactions with tissue such as polarization gating spectroscopy have suggested that polarized light can be utilized to probe shallow penetration depths in biological material and that by varying the polarization state between circular and linear polarized light, specific sampling depths can be achieved [8,9,10]. Structural color in *Pollia condensata* has been determined to be caused by Bragg reflection of helicoidally stacked cellulose microfibrils, reflecting either left or right circularly polarized light [11]. This suggests that utilizing polarized light may be able to infer physical structures in fruits.

Aquaphotomics is a recent scientific discipline conceptualized by Professor Dr Roumiana Tsenkova that utilizes water as a ‘detector’ for scientifically investigating changes associated with water structures and improving NIRS predictions [12]. For example, using the aquaphotomics approach, temperature changes in water have been identified leading to improved NIRS apple juice SSC predictions, by correcting for temperature using the method of extended multiplicative scatter correction (EMSC) [13]. Water is the major constituent of fruits but other compounds, with active OH functional groups, such as sugars, starch, and cellulose, contribute to the overall pattern of water absorption peaks in the NIR range. The second overtone region of the OH stretch, which gives rise to the strong water peak around 970 nm, is of major interest to the use of NIRS for non-destructive measurement of fruit properties [14,15].

The objective of this study was to investigate the effects of polarized and unpolarized light on unpeeled (intact) and peeled (sliced) yellow-fleshed kiwifruit (*Actinidia chinensis* var. *chinensis* ‘Zesy002′) in the second overtone region of the OH stretch of water, using aquaphotomics. Previous studies involving the removal of apple skin resulted in an improvement of prediction models for SSC and firmness, probably because of removal of the highly scattering skin [16]. By removing kiwifruit skin, it is assumed that scattering effects will reduce considerably. Sugar molecules such as sucrose are optically active and rotate the plane of linearly polarized light, which is why the effects of SSC measured on kiwifruits are investigated using linearly polarized and unpolarized light [17].

## 2. Materials and Methods

**Figure 1:**
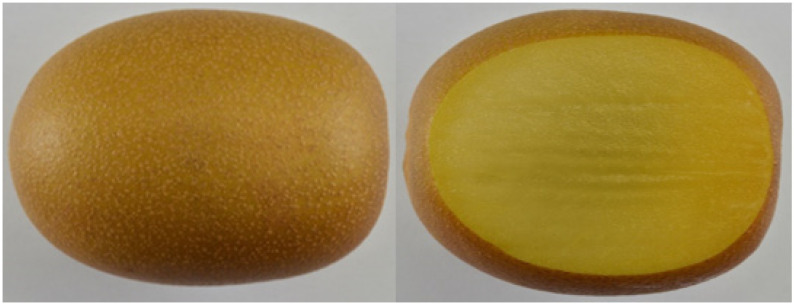
Example of unpeeled (**Left**) and peeled (**Right**) kiwifruit.

**Figure 2:**
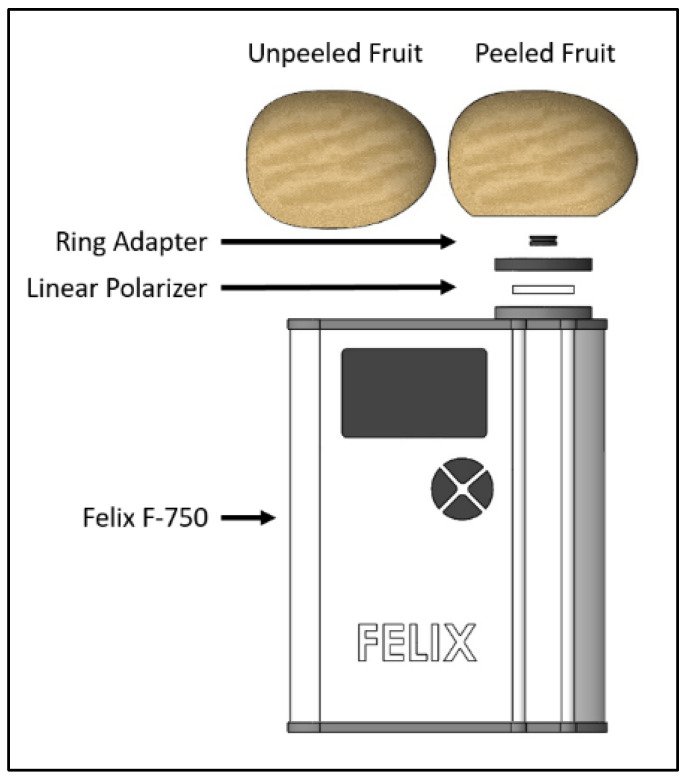
An illustration of the measurement of set up depicting peeled and unpeeled kiwifruit, a ring adapter, a linear polarizer and a Felix F-750.

Unpeeled (intact) and peeled (cut) kiwifruit (Figure 1) were placed directly on top of the ring adapter, enabling an interactance mode NIRS measurement to be made.The measurement setup (Figure 2) consisted of a bespoke ring adapter that ensured only diffuse transmitted light was collected, a linear polarizer (LPNIRE100-B, Thorlabs Inc., Newton, NJ, USA) and a handheld NIR instrument (F-750, Felix Instruments Oregon, Camas, WA, USA). 

### 2.1. Sample Preparation

Two hundred yellow-fleshed kiwifruit, sourced from a local kiwifruit packhouse (Te Puke, New Zealand), were stored at room temperature (22 ± 1 °C). Forty fruits were measured each day for five days and all fruit, except Day 1 fruit, were subjected to 100 ppm ethylene for 24 h to accelerate ripening. Using the F-750 instrument (Oregon, Camas, WA, USA), fruit was measured under four different configurations: Unpeeled Unpolarized (UU), Unpeeled Polarized (UP), Peeled Unpolarized (PU) and Peeled Polarized (PP). Unpeeled and peeled refer to intact and sliced fruit, respectively, where sliced fruit had two 2 mm slices sectioned off using a commercial meat slicer (WFS30MGB3, Wedderburn, New Zealand) to expose internal fruit tissue. After slicing, the sections of each fruit were re-placed onto the cut surface, with the whole fruit then being wrapped in cling wrap (Gladwrap^®^, Plant Based Cling Wrap, Sydney, Australia) to reduce moisture loss.

### 2.2. Spectra Collection

The linear polarizer selectively transmits an electromagnetic wave that oscillates along a defined plane and absorbs the rest. Light scattered back towards the detector consists of two components: light having undergone few scattering events and multiply scattered light [18]. The polarizer in this configuration measures light of the same input polarization state and half of the depolarized light. Only co-polarized light was measured in the polarized light configurations,

(1) $$I_{co-pol}\left(\lambda \right)=I_{\left|\right|}\left(\lambda \right)+I_{⊥}\left(\lambda \right),\;$$

where $I_{\left|\right|}\left(\lambda \right)$ refers to light having undergone a few scattering events and $I_{⊥}\left(\lambda \right)$ refers to depolarized light that has undergone multiple scattering events.

All measurements were made at room temperature (22 ± 1 °C) with the F-750 recording the average of five scans on one side of a kiwifruit. This process was repeated four times at the same location, once for each configuration. Each fruit was orientated with its stem end along the transmission axis of the linear polarizer. A separate scan was recorded for each configuration using a reflectance standard (Spectralon SRS-99-020, Labsphere, North Sutton, NH, USA) and a custom reference holder.

### 2.3. Destructive Measurements

After measurements were complete, DMC (% FW) and SSC (°Brix) were destructively measured. For DMC, an approximately 3 mm thick kiwifruit cross-sectional slice was sectioned off from the equatorial middle of each fruit. The kiwifruit slice was weighed, then dried for 24 h at 65 °C to remove moisture. The weight of the dried kiwifruit slice was then measured and the DMC was calculated as the ratio of the dry to wet weight.

For SSC, a refractometer (PAL-1, Atago Co. Ltd., Tokyo, Japan) was referenced to 0°Brix using Milli-Q water. Stem and blossom endcaps were sectioned off, about 5 mm in from the ends, and approximately 0.5 mL of juice was squeezed into the digital refractometer. The final SSC (°Brix) value was obtained by averaging the measurements from both ends of the kiwifruit.

### 2.4. Multivariate and Aquaphotomics Analysis

The fruit interactance measurements, normalized using the reference scans, were transformed to absorbance data (i.e., base 10 logarithm). The subsequent analysis used pre-processing techniques to identify key differences in measurements made in the different configurations. Pre-processing methods used on the absorbance data were:

- Mean centering;
- Standard Normal Variate (SNV);
- SNV followed by second derivative processing (SNV + 2D). The second derivative was calculated using the Savitzky–Golay method with a filter window width of 7 nm and second order polynomial smoothing. A filter width of seven was chosen as higher widths may ‘wash’ out any interesting features, and to improve the resolution.

Principal Components Analysis (PCA) analysis was carried out using PLS toolbox version 8.6.2 (Eigenvector Research Inc., Manson, WA, USA) operating under MATLAB version 2021a (MathWorks Inc., Natick, MA, USA). PCA is a useful tool that can reduce dimensionality and simplify interpretation of all kiwifruit absorbance for each configuration [19]. PCA is also useful for identifying wavelengths of interest between configurations. Loading and score plots of the first three principal components were used to explain variances for all configurations.

Aquaphotomics analysis utilizes 12 wavebands, called the water matrix coordinates (WAMACS), which are sensitive to perturbations of the water structure. The WAMACS in the range of 800–1100 nm, corresponding to the second overtone of water, were calculated using the anharmonic oscillator model and the WAMACS wavebands known in the first overtone region around 1450 nm [20]. Each WAMACS waveband represents a combination of water vibrational states and different water species, namely S0, S1, S2, S3 and S4, where the number refers to the number of hydrogen bonds. Owing to the limited optical bandwidth of the F-750 instrument, exact wavelengths provide only an indication of wavebands that are important when differentiating between configurations. Specific wavelengths were determined using:

- SNV + 2D absorbance spectra of all configurations, noting any features.
- Average SSC difference spectra (SNV + 2D absorbance) between the Low, Medium and High SSC groups.
- Principal components analysis (PCA) of different configurations, including loading and score plots.

Once selected, WAMACS wavelengths used for aquagrams were generated to visualize water perturbations using a relative standard normal variate transformation:

(2) $$A_{\lambda }^{\prime }=\frac{\left(A_{\lambda }-\mu_{\lambda }\right)}{\sigma_{\lambda }},\;$$

where $A_{\lambda }$ is the pre-processed absorbance value for a sample at wavelength λ, and μ and σ are the mean and standard deviation of the pre-processed absorbance values across all configurations at that specific wavelength. Aquagrams were then generated using a spider plot function available in MATLAB [21].

## 3. Results and Discussion

Table 1 shows the variation in SSC for all 200 kiwifruit. Fruits were assigned to Low (7–11.9°Brix), Medium (12.1–14.9°Brix) or High (15–20.5°Brix) SSC groups to compare results across all configurations at different average SSC. Total SSC varied from 7 to 20.5°Brix, with a standard deviation of 2.9°Brix.

### 3.1. Raw and SNV Spectra

**Figure 3:**
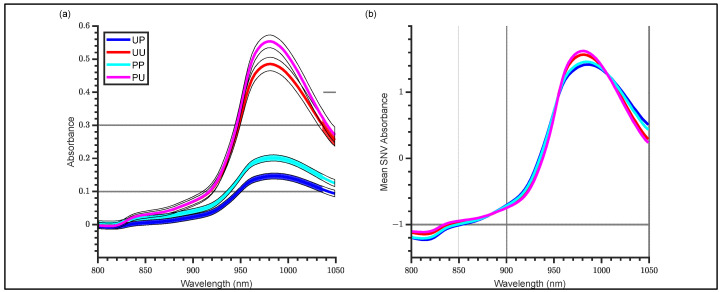
Absorbance spectra (800–1050 nm) of kiwifruit for unpeeled polarized (blue), unpeeled unpolarized (red), peeled polarized (cyan) and peeled unpolarized (magenta) for (**a**) Raw absorbance with filled areas representing mean ± std and (**b**) Mean SNV absorbance for each configuration.

Raw absorbance spectra of all kiwifruit are shown in Figure 3 in the range 800–1050 nm, where the filled areas for each configuration represent mean ± standard deviation at each wavelength. For the peeled fruit (skin removed), the absorbance increased, relative to the unpeeled fruit, for both the linearly polarized and unpolarized light and there was a substantial increase in absorbance (decreased light intensity) when using the linear polarizer (Figure 3). This demonstrates that removal of skin increases absorbance for linearly polarized and unpolarized light and that a substantial intensity, reduction is observed when using a linear polarizer. The increase in absorbance after peeling was similar to that previously observed on peeled apples [16]. The SNV transformation of the spectra also revealed clear separation between the linearly polarized and unpolarized configurations, with the former exhibiting slight broadening of the water peak compared with the latter. This can be related to the lower penetration depth of polarized light compared with unpolarized light, because of fewer interactions within the fruit tissue [22].

### 3.2. WAMACS Wavelength Selection

**Figure 4:**
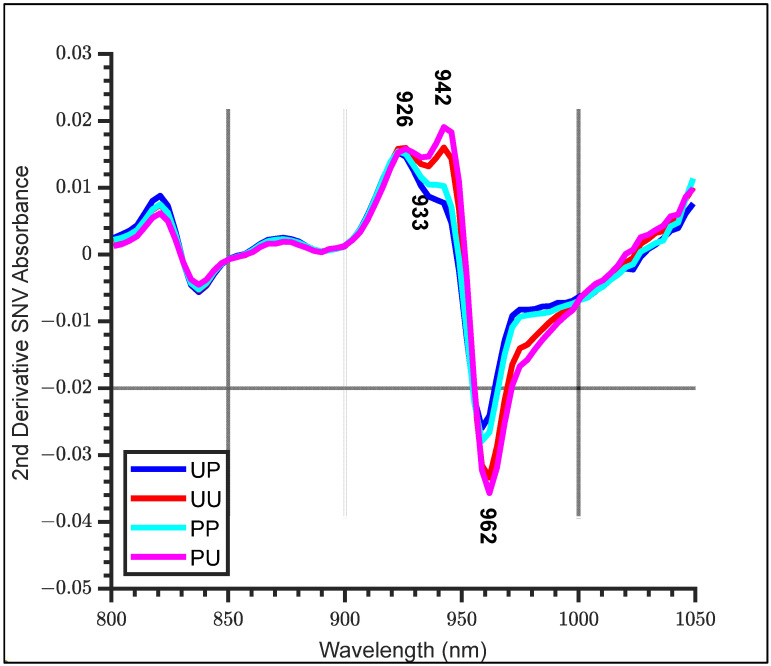
Standard Normal Variate (SNV) + 2D absorbance for each configuration (Blue—Unpeeled Polarized, Red—Unpeeled Unpolarized, Cyan—Peeled Polarized and Magenta—Peeled Unpolarized kiwifruit) in the range of 800–1050 nm.

Taking a second derivative of the SNV absorbance (SNV + 2D) revealed further differences between co-polarized and unpolarized light measurements (Figure 4). One feature included a prominent reduction in the second derivative absorbance from unpolarized to polarized light at 942 nm. Another was an apparent flattening of the linearly polarized light compared with unpolarized light from 970 to 1000 nm.

In general, the peeled configurations exhibited higher second derivative absorbance at 942 nm and lower absorbance from 960 to 1000 nm than unpeeled configurations. Kiwifruit skins are complex structures, made of largely dry dead fruit cells that will be highly scattering and result in light losses that contribute to relatively higher absorbance measurements on the unpeeled fruit compared to on the peeled fruit [23].

**Figure 5:**
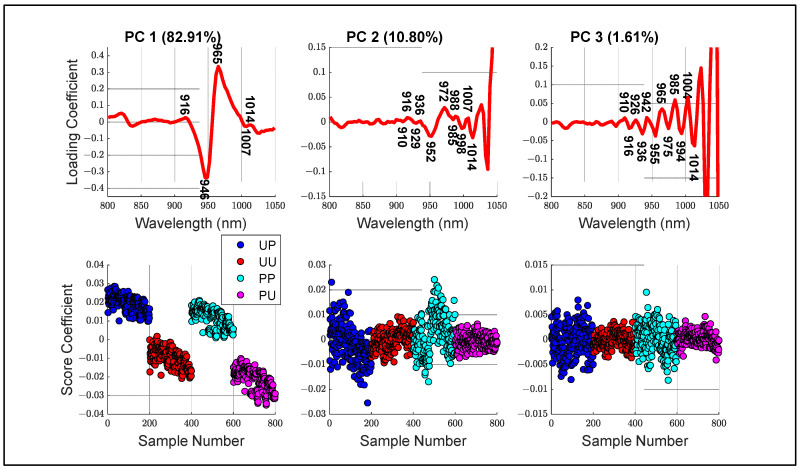
Principal Component Analysis (PCA) loading and score plots with variance explained at the top of each loading plot. For score plots, Blue (Unpeeled Polarized, 1–200), Red (Unpeeled Unpolarized, 201–400), Cyan (Peeled Polarized, 401–600) and Magenta (Peeled Unpolarized, 601–800 kiwifruit) are shown.

PCA loading and score plots for SNV and second derivatives are shown in Figure 5, where just over 95% of the variance was captured by the first three principal components (PC1—82.91%, PC2—10.80% and PC3—1.61%). Each score plot is arranged in order of Unpeeled Polarized, Unpeeled Unpolarized, Peeled Polarized, and Peeled Unpolarized (Blue, Red, Cyan and Magenta, respectively) with every 200 sample numbers indicating a new configuration.

For each principal component plot, maxima and minima wavelengths in the range of 900 to 1020 nm were identified for use with the aquaphotomics analysis. There was a clear separation between the polarized and unpolarized configurations in PC1, where there was a slight tendency for peeled configurations to shift negatively. This appears to be due to a negative correlation at 946 nm, a wavelength corresponding to a free water state ($S_{0}$), described as water molecules with a free ${\mathrm{OH}}^{-}$ functional groups. Separation between polarization states shifted positively at 965 nm, a wavelength corresponding to water molecules bonded with one hydrogen bond. Trends with the higher PCs were not so obvious.

**Figure 6:**
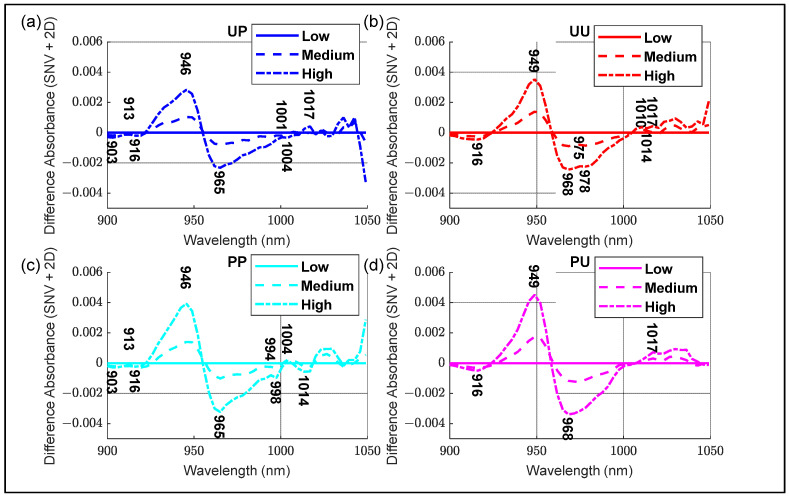
Standard Normal Variate (SNV) + 2D Soluble Solids Content (SSC) Difference Spectra for each configuration. (**a**) Unpeeled Polarized, (**b**) Unpeeled Unpolarized, (**c**) Peeled Polarized and (**d**) Peeled Unpolarized kiwifruit. Low, medium and high designations are represented by solid, dashed and dashed-dot lines, respectively.

Average SSC difference spectra (SNV + 2D absorbance) were calculated for each configuration (Figure 6) by subtracting the average spectra for all groups from the average spectra of the Low SSC group. The changes for the polarized light configurations were at 946 and 965 nm, an increase and decrease in SNV + 2D absorbance, respectively, as SSC increased. The unpolarized light configurations had similar changes occurring at 949 nm and 968 nm with the unpeeled configurations having extra features at 975 nm and 978 nm, possibly caused by the presence of the skin. All minor maxima and minima in each configuration were considered in the subsequent aquaphotomics analysis.

### 3.3. Aquaphotomics Analysis

A summary of the important WAMACS coordinates is shown in Table 2, the water bands based on calculations using the anharmonic oscillator model for the second overtone of water [20]. C1 to C5 are related to free water species and C6 to C12 are related to bound water species. Using PCA results (Figure 5), activated wavelengths, from within each band, were identified at 916 nm (C2) from PC1, PC2 and PC3; 946 nm (C5) from PC1; 965 nm (C7) from PC1; 988 nm (C9) from PC2; 1007 nm (C11) and 1014 nm (C12) from PC1 and PC2. Using the SNV + 2D spectra (Figure 4), activated wavelengths were identified at 926 nm (C3), 933 nm (C4) and 962 nm (C6). From the average SSC difference spectra (Figure 6), activated wavelengths were identified at 903 nm (C1), 975 nm (C8) and 994 nm (C10).

### 3.4. Aquagrams

#### 3.4.1. Aquagram for Polarized and Unpolarized Light

**Figure 7:**
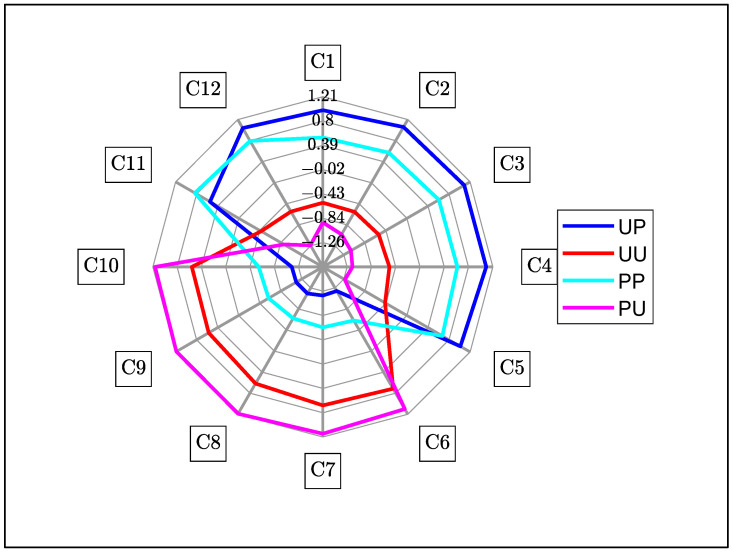
Aquagram for all configurations. Blue—Unpeeled Polarized, Red—Unpeeled Unpolarized, Cyan—Peeled Polarized and Magenta—Peeled Unpolarized kiwifruit.

The overall aquagram of all configurations (Figure 7) suggests that polarized light has relatively greater absorbance at free water states (C1 to C5) than unpolarized light, which relatively favors bound water states (C6 to C10). The exception is the bound water states at C11 and C12, where polarized light configurations exhibited relatively greater absorbance than unpolarized light. C11 and C12 are closely related to strongly bonded hydrogen water molecules so it is possible that these water structures could be polarization dependent but, as such, there is no explanation on why absorbance of C12 for unpeeled polarized is higher than peeled polarized configuration and vice versa for C11 [19]. Peeled configurations exhibited relatively greater absorbance for bound water states than the unpeeled configurations.

Polarized light is quickly depolarized in biological material such as fruit tissues, due to multiple scattering [8]. Light undergoes multiple scattering events to become entirely diffuse and depolarized (isotropically scattered) in a distance of the order of 1 mm in fruit tissue [24]. Hence, water structure interactions of polarized light are expected to be similar to unpolarized light after a penetration depth of approximately 1 mm due to such depolarization. Differences observed between polarized and unpolarized are, thus, attributable to the near-surface region of fruit and the relatively shorter pathlength of polarized light [22]. There is also potential for polarization-sensitive structures such as cellulose and starch causing differences between unpolarized and linearly polarized light. Starch granules in particular produce Maltese cross patterns when placed between crossed polarizers, a common phenomenon with birefringent material [25]. The second overtone of water contains mixed absorbance from all these structures, meaning that it may be difficult to differentiate differences between polarized and unpolarized through conventional means [14].

#### 3.4.2. Aquagram for Soluble Solids Content

**Figure 8:**
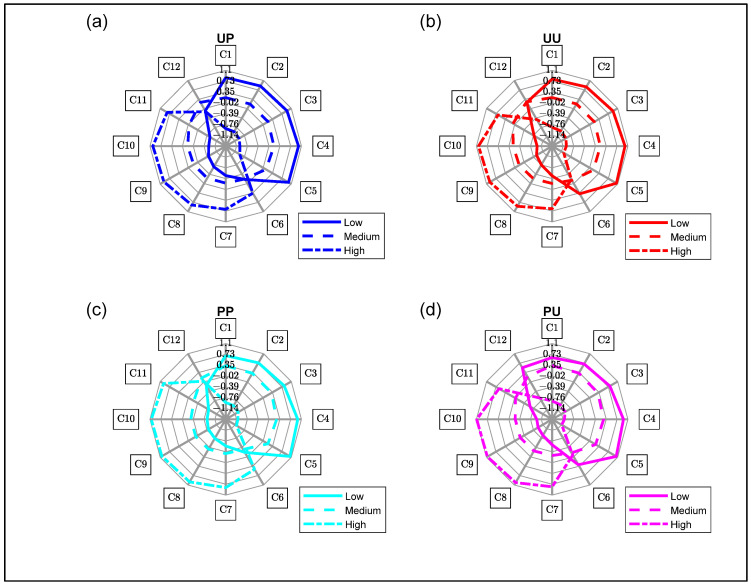
Aquagram for all configurations with different kiwifruit SSC (low, medium and high) for (**a**) Unpeeled Polarized, (**b**) Unpeeled Unpolarized, (**c**) Peeled Polarized and (**d**) Peeled Unpolarized. Solid Line—Low SSC, Dashed Line—Medium SSC and Dashed-Dot Line—High SSC.

Similar aquagrams patterns are produced for all configurations irrespective of the SSC grouping (Figure 8). Alignment with the free water species (C1 to C5) decreased, with the bound water species (C6 to C11) alignment increasing, as the average SSC increased. This is similar to previous work involving SSC grouping [20]. The aquagram suggests that differences between polarized and unpolarized responses were not related to the SSC. There is no explanation why, but it is worth noting that at C6, from the Low to High SSC group, the relative absorbance decreased for unpolarized light whereas it stayed the same for polarized configurations.

## 4. Conclusions

Two hundred yellow-fleshed kiwifruit were spectrally measured, in the NIR range from 800 to 1050 nm, under four different combinations of unpeeled and peeled fruit, and linearly polarized and unpolarized light. Clear differences in the absorbance patterns were observed between the polarized and unpolarized light, but less so between the unpeeled and peeled fruit. Differences in fruit SSC did not appear to be a significant factor associated with these differences. Under an aquaphotomics framework, there was the suggestion that bound water states absorbed more unpolarized light than polarized light. It is speculated that differences are due to polarization sensitive structures, particularly in the near surface layers, and further work is needed to investigate and explain these differences in response due to the polarization state of light.

## Figures and Tables

**Table 1:** Variation of SSC by different SSC level grouping (Low, Medium, High).

SSC Group	SSC (°Brix)	Average (°Brix)
Low SSC (*N* = 61)	7–11.9	10.4
Medium SSC (*N* = 69)	12.1–14.9	13.5
High SSC (*N* = 70)	15–20.5	16.9
All SSC (*N* = 200)	7–20.5	13.7

**Table 2:** Assignment of water absorbance bands for second overtone of water using the anharmonic oscillator model [20].

WAMACS	Assignment	Water Bands (nm)	Activated Wavelengths (nm)
C1	v3—asymmetric stretching vibration	900–908	903
C2	OH stretch—(water solvation shell)	916–920	916
C3	$v1+v3—H_{2}O$ symmetric stretching and asymmetric stretching vibration	923–927	926
C4	OH stretch (water solvation shell)	930–935	933
C5	S0 (free water)	942–955	946
C6	$\mathrm{Water}\;\mathrm{hydration},\;H_{5}O_{2}$	957–963	962
C7	S1—Water molecules with one hydrogen bond	965–973	965
C8	$v2+v3—H_{2}O$ bending andasymmetric stretching vibration	975–979	975
C9	S2—Water molecules with 2 hydrogen bonds	982–989	988
C10	S3—Water molecules with 3 hydrogen bonds	992–998	994
C11	S4—Water molecules with 4 hydrogen bonds	998–1007	1007
C12	Strongly bonded water (v1, v2)	1014–1021	1014

## Data Availability

The data presented in this study are available on request from the corresponding author.

## Sample Availability

Samples of the compounds are not available from the authors.

## References

[1] Abbott J.A. (1999). Quality measurement of fruits and vegetables. Postharvest Biol. Technol..

[2] Schaare P., Fraser D. (2000). Comparison of reflectance, interactance and transmission modes of visible-near infrared spectroscopy for measuring internal properties of kiwifruit (*Actinidia chinensis*). Postharvest Biol. Technol..

[3] Nicolaï B.M., Beullens K., Bobelyn E., Peirs A., Saeys W., Theron K.I., Lammertyn J. (2007). Nondestructive measurement of fruit and vegetable quality by means of NIR spectroscopy: A review. Postharvest Biol. Technol..

[4] Xie L., Wang A., Xu H., Fu X., Ying Y. (2016). Applications of near-infrared systems for quality evaluation of fruits: A review. Trans. ASABE.

[5] Nguyen-Do-Trong N., Dusabumuremyi J.C., Saeys W. (2018). Cross-polarized VNIR hyperspectral reflectance imaging for non-destructive quality evaluation of dried banana slices, drying process monitoring and control. J. Food Eng..

[6] Nassif R., Pellen F., Magné C., Le Jeune B., Le Brun G., Abboud M. (2012). Scattering through fruits during ripening: Laser speckle technique correlated to biochemical and fluorescence measurements. Opt. Express.

[7] Sarkar M., Gupta N., Assaad M. (2019). Monitoring of fruit freshness using phase information in polarization reflectance spectroscopy. Appl. Opt..

[8] Tuchin V.V. (2016). Polarized light interaction with tissues. J. Biomed. Opt..

[9] Gomes A.J., Turzhitsky V., Ruderman S., Backman V. (2012). Monte Carlo model of the penetration depth for polarization gating spectroscopy: Influence of illumination-collection geometry and sample optical properties. Appl. Opt..

[10] Da Silva A., Deumie C., Vanzetta I. (2012). Elliptically polarized light for depth resolved optical imaging. Biomed. Opt. Express.

[11] Vignolini S., Rudall P.J., Rowland A.V., Reed A., Moyroud E., Faden R.B., Baumberg J.J., Glover B.J., Steiner U. (2012). Pointillist structural color in Pollia fruit. Proc. Natl. Acad. Sci. USA.

[12] Muncan J., Tsenkova R. (2019). Aquaphotomics—From Innovative Knowledge to Integrative Platform in Science and Technology. Molecules.

[13] Kaur H., Künnemeyer R., McGlone A. (2020). Investigating aquaphotomics for temperature-independent prediction of soluble solids content of pure apple juice. J. Near Infrared Spectrosc..

[14] Osborne B.G., Fearn T., Hindle P.H. (1993). Practical NIR Spectroscopy with Applications in Food and Beverage Analysis.

[15] McGlone V.A., Jordan R.B., Seelye R., Martinsen P.J. (2002). Comparing density and NIR methods for measurement of kiwifruit dry matter and soluble solids content. Postharvest Biol. Technol..

[16] Lu R., Guyer D.E., Beaudry R.M. (2000). Determination of firmness and sugar content of apples using near-infrared diffuse reflectance. J. Texture Stud..

[17] Compton R., Mahurin S., Zare R.N. (1999). Demonstration of optical rotatory dispersion of sucrose. J. Chem. Educ..

[18] Gobrecht A., Bendoula R., Roger J.-M., Bellon-Maurel V. (2015). Combining linear polarization spectroscopy and the Representative Layer Theory to measure the Beer–Lambert law absorbance of highly scattering materials. Anal. Chim. Acta.

[19] Tsenkova R., Munćan J., Pollner B., Kovacs Z. (2018). Essentials of aquaphotomics and its chemometrics approaches. Front. Chem..

[20] Kaur H. (2020). Investigating Aquaphotomics for Fruit Quality Assessment. Ph.D. Thesis.

[21] (2021). Moses. Spider_Plot. Moses. GitHub. https://github.com/NewGuy012/spider_plot/releases/tag/16.6.

[22] Liu Y., Kim Y.L., Li X., Backman V. (2005). Investigation of depth selectivity of polarization gating for tissue characterization. Opt. Express.

[23] Redgwell R.J., MacRae E., Hallett I., Fischer M., Perry J., Harker R. (1997). In vivo and in vitro swelling of cell walls during fruit ripening. Planta.

[24] Fang Z.-h., Fu X.-p., He X.-m. (2016). Investigation of absorption and scattering characteristics of kiwifruit tissue using a single integrating sphere system. J. Zhejiang Univ.-Sci. B.

[25] Tongdang T. (2008). Some properties of starch extracted from three Thai aromatic fruit seeds. Starch-Stärke.

